# 

**Published:** 1880-01-20

**Authors:** 


Entered at the Post-Office at Atlanta as second-class matter.

VOL. X. JANUARY 20, 1880. NO. 1.

### THE SOUTHERN MEDICAL RECORD:

### A MONTHLY JOURNAL OF PRACTICAL MEDICINE.

Terms: $2 00 per Annum, in Advance; 4 Copies $6.00; Single Copies 20 Cents.

*"QUICQUID PRÆCIPIES ESTO BREVIS."*

Address R. C. WORD, M.D., Managing Editor, Atlanta, Ga.

